# Author Correction: Unbinned contigs expand known diversity in the global microbiome

**DOI:** 10.1038/s41564-026-02354-y

**Published:** 2026-04-27

**Authors:** Vishnu Prasoodanan PK, Oleksandr M. Maistrenko, Anthony Fullam, Daniel R. Mende, Ece Kartal, Luis Pedro Coelho, Anja Spang, Peer Bork, Thomas S. B. Schmidt

**Affiliations:** 1 APC Microbiome and School of Medicine, University College Cork, Cork, Ireland; 2 Department of Marine Microbiology and Biogeochemistry, Royal Netherlands Institute for Sea Research, Texel, the Netherlands; 3 Molecular Systems Biology Unit, European Molecular Biology Laboratory, Heidelberg, Germany; 4 Human Biology-Microbiome-Quantum Research Center (WPI-Bio2Q), Keio University, Tokyo, Japan; 5 Institute for Computational Biomedicine, Faculty of Medicine, University of Heidelberg, Heidelberg, Germany; 6 Centre for Microbiome Research, School of Biomedical Sciences, Queensland University of Technology, Translational Research Institute, Brisbane, Queensland Australia; 7 Max Delbrück Center for Molecular Medicine, Berlin, Germany; 8 Department of Bioinformatics, Biocenter, University of Würzburg, Würzburg, Germany; 9 Present Address: Swammerdam Institute for Life Sciences, University of Amsterdam, Amsterdam, the Netherlands

**Keywords:** Classification and taxonomy, Environmental microbiology, Metagenomics, Metagenomics, Evolutionary ecology

Correction to: *Nature Microbiology* 10.1038/s41564-026-02314-6, published online 3 April 2026.

This article was initially published under a Creative Commons CC BY 4.0 license, © the Author(s), and is now available under a Creative Commons CC BY 3.0 IGO license, © European Molecular Biology Laboratory and the Author(s).

