# Supplementary material for: Unbinned contigs expand known diversity in the global microbiome
Source: Nat Microbiol. 2026 Apr 3;11(5):1437–49. doi: 10.1038/s41564-026-02314-6 (PMC13171631; doi:10.1038/s41564-026-02314-6)
Supplement: Supplementary file 1 — Supplementary Figs. 1 and 2 and discussion. [file 41564_2026_2314_MOESM1_ESM.pdf]

nature microbiology

Supplementary information https://doi.org/10.1038/s41564-026-02314-6

Unbinned contigs expand known diversity in the global microbiome

In the format provided by the authors and unedited

Supplementary Discussion

Biological signal and noise among singleton marker gene clusters.

A pertinent question for all marker gene-based surveys of (microbial) diversity is *accuracy*. An ideal approach is both *sensitive* and *precise*: it recovers 'real' biological signal (i.e., sequence variants representing real biological lineages) with low rates of both false negatives (real sequences that are missed) and false positives (erroneous calls, due to noise introduced e.g. by sequence artifacts).

Amplicon-based surveys arguably provide only limited accuracy. Amplification bias can introduce false positives based on 'chimeric' artifacts, but the use of different primers (with differential taxonomic coverage) on different gene subregions introduces more complex skews [1]. Moreover, definitions of similarity cutoffs for 'species-level' resolution in 16S rRNA remain debated, and different lineages may indeed share identical amplified subsequences [2]. Finally, the focus on just one taxonomic marker prohibits links to functional or genomic context for uncultivated lineages.

An integrated survey across multiple marker genes based on metagenomic assemblies mitigates most of these issues, yet may introduce others. In particular, a major fraction of observed (species-level) marker gene clusters were *singletons*, i.e. containing only one sequence. Below, we discuss to what extent these singleton clusters may be due to noise (i.e., artifacts introduced during data generation and processing) and why we are confident that most indeed represent 'real' biological lineages.

Possible assembly artifacts. A recent benchmark estimated metagenomic misassembly errors to be in the range of ~7% of contigs [3]. Assembly errors are not randomly distributed and most are expected to be either (i) intra-species chimerae (which would not greatly affect species-level clustering) or (ii) of such a type that HMMs would no longer detect a (partial) gene of sufficient length to pass our filters [4]. In a large-scale benchmark of ~300M assembled ORFs as part of the Global Microbial Gene Catalogue study [5], 75% of singleton genes were detectable in multiple metagenomic samples based on raw reads; 92% fell into known gene families; and only 0.4% were potential chimeras based on very inclusive criteria. We therefore estimate that misassembled genes with gene cluster-breaking errors are rare in our analysis.

Singleton gene clusters are not disproportionately overrepresented among unbinned contigs. Extended Figure 4 shows that the fraction of singleton clusters derived from MAGs and unbinned contigs is higher than for reference genomes (proGenomes3), but not disproportionately so. Indeed, a significant fraction of (singleton) clusters among reference genomes was also not detected among our metagenomic assemblies. In our analyses on clade size distributions (Willis / Yule curves, Figure 4), the estimated 'rho' values (Yule-Simon shape parameter) for Archaea and Bacteria are lower for SPIRE-derived data (including unbinned contigs) than for GTDB or GBIF (Global Biodiversity Information Facility) archaeal and bacterial reference taxonomies, as well as than for 16S-based OTUs from the Microbe Atlas Project. Among other things, lower 'rho' values correspond to size distributions that are less dominated by

singletons and doubletons. In other words, SPIRE-derived clade size distributions are broadly in line with reference databases, but if anything, they are less heavy on singleton and small clades.

Possible underestimation of diversity due to alternate genetic codes. Our study does not explicitly account for alternative genetic codes; our ORF calls (and HMM searches) were conducted per metagenomic sample and gene callers perform poorly at detecting alternate codes (see e.g. [6] or [7]), so it is likely that a significant number of real ORFs were prematurely truncated which would lead to an overall *under*-estimation of diversity.

Taken together, we are confident that most singleton marker gene clusters in our data correspond to real biological entities, rather than technical or data artefacts, and that diversity estimates excluding singleton clusters represent a conservative lower bound.

Soils and aquatic habitats remain major reservoirs of unexplored microbial diversity.

Although our dataset contained 53,949 gastrointestinal metagenomes (corresponding to 59% of the total), these accounted for just ~253k (or 36%) of discoverable species for Bacteria and ~2k (or 7%) for Archaea (Extended Figure 5). This corresponds to an average of 4.7 newly added bacterial and 0.04 archaeal species per sample. Indeed, the human gut alone (across age groups, geography and disease states, yet still representing just one host species) accounted for 49% of total samples, but just 16% of discoverable bacterial and 0.8% of archaeal diversity. A further 12,098 metagenomes from non-intestinal human body sites and the built environment increased the tally to ~72% of total samples, but contributed only an additional 13k (~2% of the total, or 1.05 per sample) bacterial and 0.2k (<1%, or 0.02 per sample) archaeal species. In other words, while human-associated, gastrointestinal and built environment metagenomes represented almost three quarters of sampling effort, they only accounted for 38% of discoverable bacterial and 8% of archaeal diversity.

In contrast, plant-associated habitats represented just 1.5k samples (1.7% of the total), but accounted for 47k (6.7%) bacterial species discoverable beyond those contained in animal-associated and anthropogenic habitats, with the rhizosphere standing out as particular hotspot of bacterial diversity at 74.4 new species added per sample. Yet the largest steps in the collector's curve were observed for soils (~6% samples contributing 16% of unique bacterial and 9% of archaeal diversity, at 21.8 and 0.45 added species per sample) and aquatic habitats (~16% of samples contributing 32% of bacterial and 78% of archaeal diversity, at 15.1 and 1.4 novel species per sample). Wetlands, including e.g. salt marshes and peatlands, were further discovery hotspots for both Bacteria and Archaea (at 53.7 and 5.0 uniquely added species per sample, respectively), as were hydrothermal vents (21.5 and 6.2 species per sample). Although these and other comparatively undersampled habitats generally contributed more novelty per sample than well-represented environments (like the human gut), the overall correlation between sampling effort and newly discovered species was only moderately negative ($\rho_{Spearman}$ = -0.21 for Bacteria and -0.26 for Archaea).

Additional notes on technical caveats and sampling bias.

We note that although our methodology includes filtering and correction for various error sources, and although assembled sequences greatly underestimate the total sequence variation represented among raw metagenomic reads [5], the presence of assembly and clustering artefacts and spurious gene variants (including paralogs, or eukaryotic or viral orthologs) are possible sources of noise that may lead to an overestimation of diversity. Either way, the gap between genomically captured and unbinned discoverable diversity was growing (rather than closing) in virtually all tested habitats. And given that most metagenomically derived genes [5] and inferred gene families [6,8] are likewise unaccounted for by genomes, our results define a range of how many additional lineages may be genomically disentangled from the vast space of unbinned and unclassified reads with improved algorithms. Indeed, constantly refined sampling and sample processing protocols, the increasing use of longread technologies [9,10], faster mapping tools that render multi-sample co-binning more computationally tractable [11,12], a novel generation of binning tools [13–15] and innovations like the iterative targeted co-assembly workflow in Bin Chicken [16] promise to make major inroads into this bulk of untapped diversity.

Our dataset reflects existing sampling bias both *between* habitats (human fecal samples dominate the survey) and *within* environments (uneven coverage geographically, along depth-elevation gradients and with regard to host species) as individual datasets are usually generated for specific purposes, rather than as part of global surveys. Indeed, the habitat definitions used in the present work are – by design – pragmatic and guided by data availability, and not necessarily reflective of the underlying diversity of environmental conditions [17]: for example, soils are structured into many heterogeneous sub-environments with physicochemical and biotic parameters that vary at micrometer scale [18,19], whereas well-mixed ocean layers can present homogeneous environments across several square kilometers [20]. Moreover, while we use metagenomic samples as units of reference, we note that established protocols capture very different scopes of underlying communities: while typical ocean water samples (filtered from 10-100L [20]) and human fecal samples (~1g of feces) carry total microbial loads of 10^{10}-10^{11} cells [21], typical soil samples of 1-2g [22] contain 2-3 orders of magnitude fewer cells [23]. Given that Earth is expected to harbour a total of ~10^{29} microbial cells each in ocean water and soils, but just 10^{23}-10^{24} cells in the global human gut microbiome [23], this puts both the abscissa (i.e., the number of samples considered) and the ordinate (i.e., the number of clades discovered) in the presented rarefaction curves into further perspective. Finally, both physiological and technical factors may reduce (or bias) recall: among others, different membrane and cell wall types yield differentially to common extraction protocols [24], genomic composition (including GC content) and structure impact sequencing protocols [25], and current gene calling algorithms still often struggle with non-standard genetic codes [6]. As a consequence, we caution against extrapolating total species richness estimates from our rarefaction curves, as further discussed in the main text.

Supplementary Discussion References.

1. Abellan-Schneyder, I., Siebert, A., Hofmann, K., Wenning, M. & Neuhaus, K. Full-length SSU rRNA gene sequencing allows species-level detection of bacteria, Archaea, and yeasts present in milk. *Microorganisms* **9**, 1251 (2021).
2. Mende, D. R., Sunagawa, S., Zeller, G. & Bork, P. Accurate and universal delineation of prokaryotic species. *Nature Publishing Group* **10**, 881–884 (2013).
3. Mineeva, O. *et al.* ResMiCo: Increasing the quality of metagenome-assembled genomes with deep learning. *PLoS Comput. Biol.* **19**, e1011001 (2023).
4. Lai, S. *et al.* metaMIC: reference-free misassembly identification and correction of de novo metagenomic assemblies. *Genome Biol.* **23**, 242 (2022).
5. Coelho, L. P. *et al.* Towards the biogeography of prokaryotic genes. *Nature* **601**, 252–256 (2022).
6. Rodríguez Del Río, Á. *et al.* Functional and evolutionary significance of unknown genes from uncultivated taxa. *Nature* **626**, 377–384 (2024).
7. Shulgina, Y. & Eddy, S. R. A computational screen for alternative genetic codes in over 250,000 genomes. *Elife* **10**, (2021).
8. Vanni, C. *et al.* Unifying the known and unknown microbial coding sequence space. *Elife* **11**, (2022).
9. Sereika, M. *et al.* Oxford Nanopore R10.4 long-read sequencing enables the generation of near-finished bacterial genomes from pure cultures and metagenomes without short-read or reference polishing. *Nat. Methods* **19**, 823–826 (2022).
10. Sereika, M. *et al.* Recovery of highly contiguous genomes from complex terrestrial habitats reveals over 15,000 novel prokaryotic species and expands characterization of soil and sediment microbial communities. *Bioinformatics* (2024).
11. Shaw, J. & Yu, Y. W. Fairy: fast approximate coverage for multi-sample metagenomic binning. *Microbiome* **12**, 151 (2024).
12. Sahlin, K. Strobealign: flexible seed size enables ultra-fast and accurate read alignment. *Genome Biol.* **23**, 260 (2022).
13. Nissen, J. N. *et al.* Improved metagenome binning and assembly using deep variational autoencoders. *Nat. Biotechnol.* **39**, 555–560 (2021).
14. Pan, S., Zhao, X.-M. & Coelho, L. P. SemiBin2: self-supervised contrastive learning leads to better MAGs for short- and long-read sequencing. *Bioinformatics* **39**, i21–i29 (2023).
15. Wang, Z. *et al.* Effective binning of metagenomic contigs using contrastive multi-view representation learning. *Nat. Commun.* **15**, 585 (2024).
16. Aroney, S. T. N., Newell, R. J. P., Tyson, G. W. & Woodcroft, B. J. Bin Chicken: targeted metagenomic coassembly for the efficient recovery of novel genomes. *Nat. Methods* 1–9 (2025) doi:10.1038/s41592-025-02901-1.
17. Kim, C. Y. *et al.* Planetary microbiome structure and generalist-driven gene flow across disparate habitats. *bioRxiv* (2025) doi:10.1101/2025.07.18.664989.
18. Ruamps, L. S., Nunan, N. & Chenu, C. Microbial biogeography at the soil pore scale. *Soil Biol. Biochem.* **43**, 280–286 (2011).
19. Bach, E. M., Williams, R. J., Hargreaves, S. K., Yang, F. & Hofmockel, K. S. Greatest soil microbial diversity found in micro-habitats. *Soil Biol. Biochem.* **118**, 217–226 (2018).
20. Sunagawa, S. *et al.* Ocean plankton. Structure and function of the global ocean microbiome. *Science* **348**, 1261359 (2015).
21. Nishijima, S. *et al.* Fecal microbial load is a major determinant of gut microbiome variation and a confounder for disease associations. *Cell* **188**, 222–236.e15 (2025).
22. Bahram, M. *et al.* Structure and function of the global topsoil microbiome. *Nature* **560**, 233–237 (2018).
23. Whitman, W. B., Coleman, D. C. & Wiebe, W. J. Prokaryotes: the unseen majority. *Proc. Natl. Acad. Sci. U. S. A.* **95**, 6578–6583 (1998).
24. Costea, P. I. *et al.* Towards standards for human fecal sample processing in metagenomic studies. *Nat. Biotechnol.* **35**, 1069–1076 (2017).
25. Benjamini, Y. & Speed, T. P. Summarizing and correcting the GC content bias in high-throughput sequencing. *Nucleic Acids Res.* **40**, e72 (2012).

Figure S1. Habitat-stratified rarefaction curves for Archaea. Species discovery curves as described in the main text and shown in Figure 1, but subset by individual habitats.

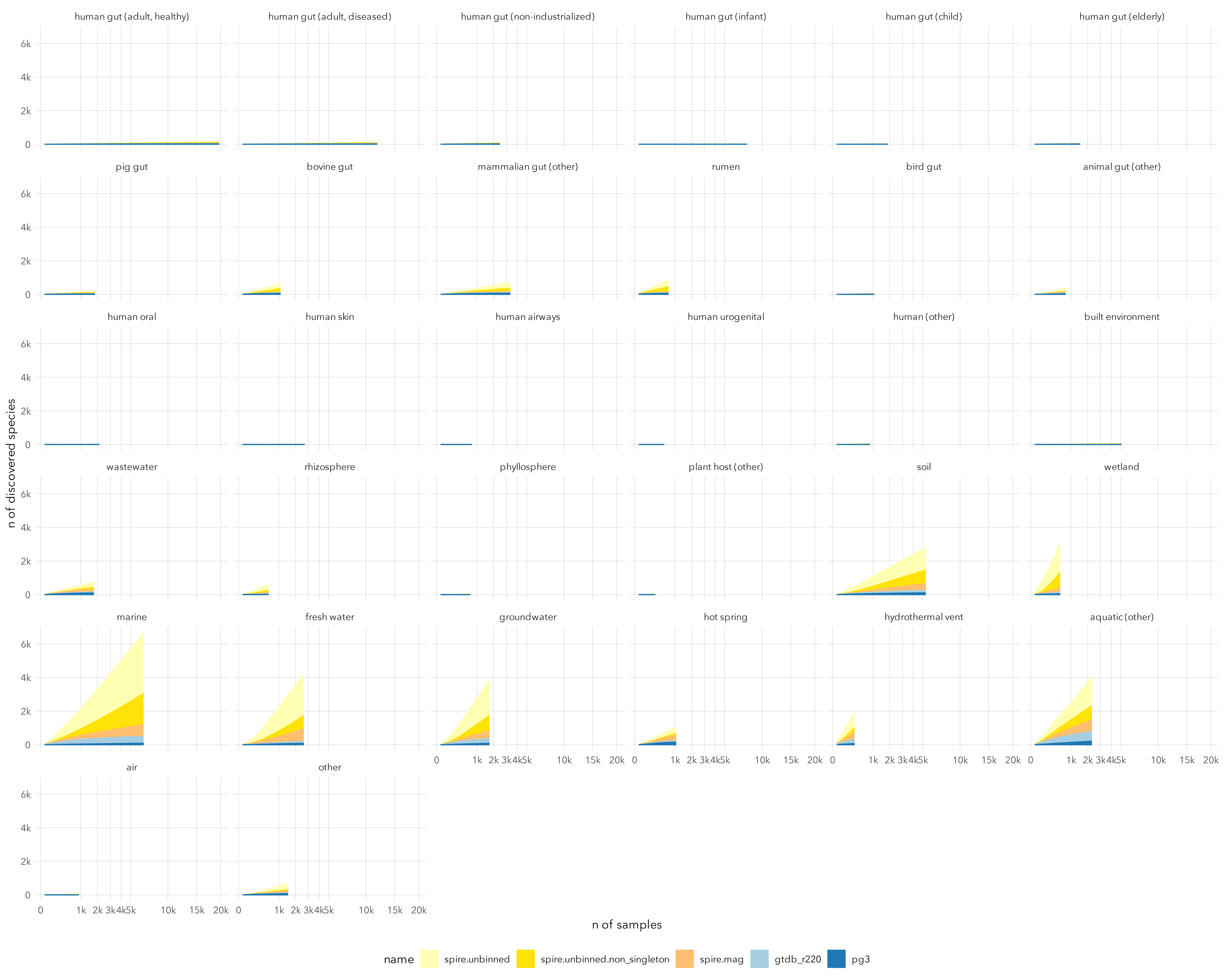
human gut (adult, healthy)
human gut (adult, diseased)
human gut (non-industrialized)
human gut (infant)
human gut (child)
human gut (elderly)
pig gut
bovine gut
mammalian gut (other)
rumen
bird gut
animal gut (other)
human oral
human skin
human airways
human urogenital
human (other)
built environment
wastewater
rhizosphere
phyllosphere
plant host (other)
soil
wetland
marine
fresh water
groundwater
hot spring
hydrothermal vent
aquatic (other)
air
other
0
2k
4k
6k
n of discovered species
0
1k
2k
3k
4k
5k
10k
15k
20k
n of samples
name
spire.unbinned
spire.unbinned.non_singleton
spire.mag
gtdb_r220
pg3

Figure S2. Habitat-stratified rarefaction curves for Bacteria. Species discovery curves as described in the main text and shown in Figure 1, but subset by individual habitats.

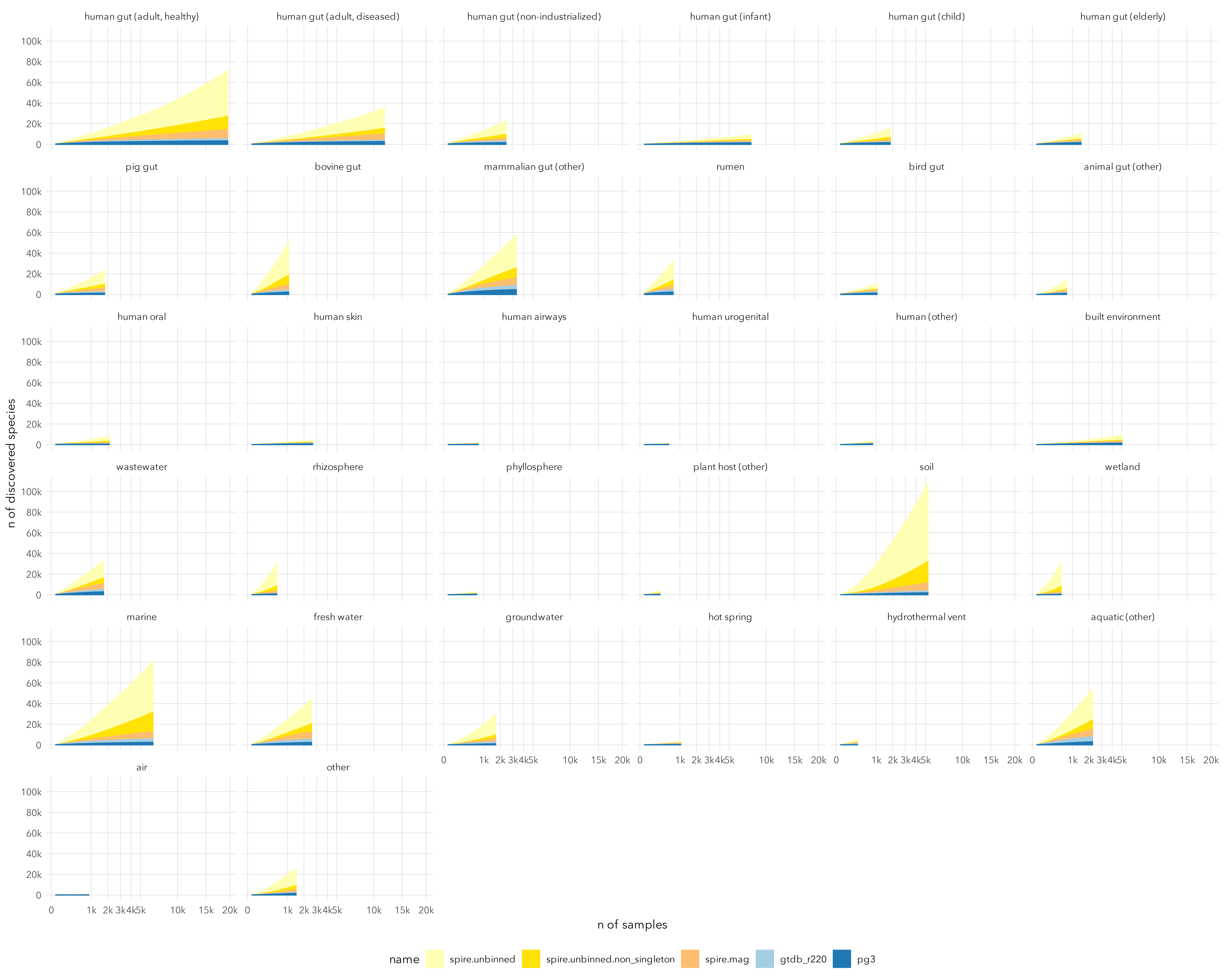

human gut (adult, healthy)
human gut (adult, diseased)
human gut (non-industrialized)
human gut (infant)
human gut (child)
human gut (elderly)
pig gut
bovine gut
mammalian gut (other)
rumen
bird gut
animal gut (other)
human oral
human skin
human airways
human urogenital
human (other)
built environment
wastewater
rhizosphere
phyllosphere
plant host (other)
soil
wetland
marine
fresh water
groundwater
hot spring
hydrothermal vent
aquatic (other)
air
other
0
20k
40k
60k
80k
100k
1k
2k
3k4k5k
10k
15k
20k
n of discovered species
n of samples
name
spire.unbinned
spire.unbinned.non_singleton
spire.mag
gtdb_r220
pg3
