# Supplementary material for: Unbinned contigs expand known diversity in the global microbiome
Source: Nat Microbiol. 2026 Apr 3;11(5):1437–49. doi: 10.1038/s41564-026-02314-6 (PMC13171631; doi:10.1038/s41564-026-02314-6)
Supplement: Supplementary file 2 — Reporting Summary [file 41564_2026_2314_MOESM2_ESM.pdf]

nature portfolio

Corresponding author(s): Schmidt, Thomas Sebastian Benedikt

Last updated by author(s): Nov 17, 2025

Reporting Summary

Nature Portfolio wishes to improve the reproducibility of the work that we publish. This form provides structure for consistency and transparency in reporting. For further information on Nature Portfolio policies, see our Editorial Policies and the Editorial Policy Checklist.

Statistics

For all statistical analyses, confirm that the following items are present in the figure legend, table legend, main text, or Methods section.

n/a	Confirmed	
☐	☒	The exact sample size (*n*) for each experimental group/condition, given as a discrete number and unit of measurement
☒	☐	A statement on whether measurements were taken from distinct samples or whether the same sample was measured repeatedly
☐	☒	The statistical test(s) used AND whether they are one- or two-sided<br>*Only common tests should be described solely by name; describe more complex techniques in the Methods section.*
☒	☐	A description of all covariates tested
☐	☒	A description of any assumptions or corrections, such as tests of normality and adjustment for multiple comparisons
☐	☒	A full description of the statistical parameters including central tendency (e.g. means) or other basic estimates (e.g. regression coefficient) AND variation (e.g. standard deviation) or associated estimates of uncertainty (e.g. confidence intervals)
☐	☒	For null hypothesis testing, the test statistic (e.g. *F*, *t*, *r*) with confidence intervals, effect sizes, degrees of freedom and *P* value noted<br>*Give P values as exact values whenever suitable.*
☒	☐	For Bayesian analysis, information on the choice of priors and Markov chain Monte Carlo settings
☒	☐	For hierarchical and complex designs, identification of the appropriate level for tests and full reporting of outcomes
☐	☒	Estimates of effect sizes (e.g. Cohen's *d*, Pearson's *r*), indicating how they were calculated

Our web collection on statistics for biologists contains articles on many of the points above.

Software and code

Policy information about availability of computer code

Data collection	SPIRE v1<br>proGenomes v3<br>GTDB r220<br>GTDB r226<br>HMMer v3.4<br>GTDB-tk v2.4.0<br>microntology v0.3<br>mapref v3.0<br>GBIF taxonomy via taxizedb package, acc. 07-2025
Data analysis	Commented analysis code was uploaded to a GitHub repository: https://github.com/grp-schmidt/ms-census<br>All external tools used for analyses are detailed, with version numbers, in the main text.<br>ttax-<br>MMSeqs2 v15.6f452<br>HMMER v3.4<br>clipkit v1.4.1<br>FastTree2 v2.1.11<br>castor v1.8.3<br>taxizedb v0.3.2

For manuscripts utilizing custom algorithms or software that are central to the research but not yet described in published literature, software must be made available to editors and reviewers. We strongly encourage code deposition in a community repository (e.g. GitHub). See the Nature Portfolio guidelines for submitting code & software for further information.

Data

Policy information about availability of data

All manuscripts must include a data availability statement. This statement should provide the following information, where applicable:

- Accession codes, unique identifiers, or web links for publicly available datasets
- A description of any restrictions on data availability
- For clinical datasets or third party data, please ensure that the statement adheres to our policy

Source data was derived from the SPIRE database (spire.embl.de) where it was prepared as described in https://doi.org/10.1093/nar/gkad943. External datasets were obtained from the proGenomes (v3) and GTDB (r220) databases, as detailed in the Methods section of the main text. Genes for analyses were extracted via HMMs (using HMMer v3) as described in the main text. No custom code was required for data collection; data curation (annotation of habitat information etc based on sample metadata) was perfomed as described in the primary SPIRE db reference and further detailed in the main text.
All data sources and tools are detailed, with version numbers and/or access dates, in the main text.

Metagenomic assemblies, Metagenome-Assembled Genomes (MAGs), gene calls and corresponding annotations are available via spire.embl.de/downloads. Extracted marker gene sequences for the ar53 and bac122 sets from SPIRE assemblies, proGenomes3 and GTDB r220 are likewise available via spire.embl.de/downloads. Pre-processed and derived data is available via Zenodo (https://zenodo.org/records/17482698). Inferred marker gene phylogenies with annotations, as well as pre-generated tree visualizations for archaeal markers are available via the EBI BioStudies repository under accessions S-BSST2111, S-BSST2112, S-BSST2113, S-BSST2116, and S-BSST2117.

Research involving human participants, their data, or biological material

Policy information about studies with human participants or human data. See also policy information about sex, gender (identity/presentation), and sexual orientation and race, ethnicity and racism.

Reporting on sex and gender	n.a.
Reporting on race, ethnicity, or other socially relevant groupings	n.a.
Population characteristics	n.a.
Recruitment	n.a.
Ethics oversight	n.a.

Note that full information on the approval of the study protocol must also be provided in the manuscript.

Field-specific reporting

Please select the one below that is the best fit for your research. If you are not sure, read the appropriate sections before making your selection.

☐ Life sciences ☐ Behavioural & social sciences ☒ Ecological, evolutionary & environmental sciences

For a reference copy of the document with all sections, see nature.com/documents/nr-reporting-summary-flat.pdf

Ecological, evolutionary & environmental sciences study design

All studies must disclose on these points even when the disclosure is negative.

Study description	A computational re-appraisal of discoverable diversity in metagenomically assembled contigs. Clustering >500M taxonomic marker gene sequences to species level, we track how many species-level groups in publicly available data are missed by common genome-centric approaches (including reference, isolate-based genomes and metagenome-assembled genomes). Moreover, based on large marker gene phylogenies, we provide estimates of how many additional deeper clades (genus to phylum) are 'hiding' in public data, but missed by current genome binning methods. Our analyses are stratified by microbial habitat and in particular provide habitat-specific 'discovery coefficients' that quantify the (differential) expectation of how many more lineages will be discovered as more metagenomic sequence data is added to the survey. Finally, we explored whether microbial clade size distributions follow empirical power laws, in line with century-old hypotheses on biodiversity.
Research sample	23.2 Tbp of assembled metagenomic contigs, sourced from the SPIRE db (spire.embl.de), from which a subset of 92k well-annotated metagenomic samples (curated public data) were selected. In addition, we analysed the reference genome databases proGenomes3 and GTDB r220 that provide (curated and processed) datasets of publicly available prokaryotic genomes. From these datasets, we extracted 502M sequences for 130 established taxonomic marker genes on which all further analyses were based.
Sampling strategy	No sample size calculations were performed. Our survey of 92k metagenomes is (to our knowledge) the largest such dataset currently available.

Data collection	Data was obtained from publicly available sources as described above under 'Research sample'.
Timing and spatial scale	N/A
Data exclusions	We excluded several taxonomic marker genes that are either known to hit orthologs across multiple domains (e.g., models that hit both archaeal and bacterial genes) or showed problematic phylogenies with signs of (erroneously included) paralogs upon manual inspection.
Reproducibility	Analyses were re-run (with modified parameters) several times and iteratively, starting from the marker gene sequence clustering step. Analysis code is available via a GitHub repository (see above); intermediary datasets / analyses are available via dedicated repositories and via spire.embl.de/downloads.
Randomization	N/A
Blinding	N/A

Did the study involve field work? ☐ Yes ☒ No

Reporting for specific materials, systems and methods

We require information from authors about some types of materials, experimental systems and methods used in many studies. Here, indicate whether each material, system or method listed is relevant to your study. If you are not sure if a list item applies to your research, read the appropriate section before selecting a response.

Materials & experimental systems

n/a	Involved in the study
☒	☐ Antibodies
☒	☐ Eukaryotic cell lines
☒	☐ Palaeontology and archaeology
☒	☐ Animals and other organisms
☒	☐ Clinical data
☒	☐ Dual use research of concern
☒	☐ Plants

Methods

n/a	Involved in the study
☒	☐ ChIP-seq
☒	☐ Flow cytometry
☒	☐ MRI-based neuroimaging

Plants

Seed stocks	*Report on the source of all seed stocks or other plant material used. If applicable, state the seed stock centre and catalogue number. If plant specimens were collected from the field, describe the collection location, date and sampling procedures.*
Novel plant genotypes	*Describe the methods by which all novel plant genotypes were produced. This includes those generated by transgenic approaches, gene editing, chemical/radiation-based mutagenesis and hybridization. For transgenic lines, describe the transformation method, the number of independent lines analyzed and the generation upon which experiments were performed. For gene-edited lines, describe the editor used, the endogenous sequence targeted for editing, the targeting guide RNA sequence (if applicable) and how the editor was applied.*
Authentication	*Describe any authentication procedures for each seed stock used or novel genotype generated. Describe any experiments used to assess the effect of a mutation and, where applicable, how potential secondary effects (e.g. second site T-DNA insertions, mosiacism, off-target gene editing) were examined.*
